# Development of a Cuttlefish-Inspired Amphibious Robot with Wave-Motion Propulsion and Rigid–Flexible Coupling

**DOI:** 10.3390/biomimetics10060396

**Published:** 2025-06-12

**Authors:** Yichao Gao, Felix Pancheri, Tim C. Lueth, Yilun Sun

**Affiliations:** Institute of Micro Technology and Medical Device Technology, Technical University of Munich, 85748 Garching, Germany; yichao.gao@tum.de (Y.G.); felix.pancheri@tum.de (F.P.); tim.lueth@tum.de (T.C.L.)

**Keywords:** amphibious robot, rigid–flexible coupling, wave-motion propulsion, biomimetic locomotion, cuttlefish-inspired robotics

## Abstract

Amphibious robots require efficient locomotion strategies to enable smooth transitions between terrestrial and aquatic environments. Drawing inspiration from the undulatory movements of aquatic organisms such as cuttlefish and knifefish, this study introduces a bio-inspired propulsion system that emulates natural wave-based locomotion to improve adaptability and propulsion efficiency. A novel mechanism combining crank–rocker and sliding components is proposed to generate wave-like motions in robotic legs and fins, supporting both land crawling and aquatic paddling. By adopting a rigid–flexible coupling design, the system achieves a balance between structural integrity and motion flexibility. The effectiveness of the mechanism is systematically investigated through kinematic modeling, animation-based simulation, and experimental validation. The developed kinematic model captures the principles of wave propagation via the Crank–Slider–Rocker structure, offering insights into motion efficiency and thrust generation. Animation simulations are employed to visually validate the locomotion patterns and assess coordination across the mechanism. A functional prototype is fabricated and tested in both terrestrial and aquatic settings, demonstrating successful amphibious locomotion. The findings confirm the feasibility of the proposed design and underscore its potential in biomimetic robotics and amphibious exploration.

## 1. Introduction

Amphibious robots have attracted significant attention due to their capability to operate effectively across diverse terrains, offering great potential for environmental monitoring, search and rescue, and underwater exploration. Natural species such as cuttlefish, salamanders, and mudskippers exhibit highly adaptive locomotion mechanisms that allow efficient movement in both aquatic and terrestrial environments. Inspired by these biological examples, considerable research has been dedicated to developing amphibious robots capable of seamless transitions between land and water [1,2,3].

**Figure 1:**
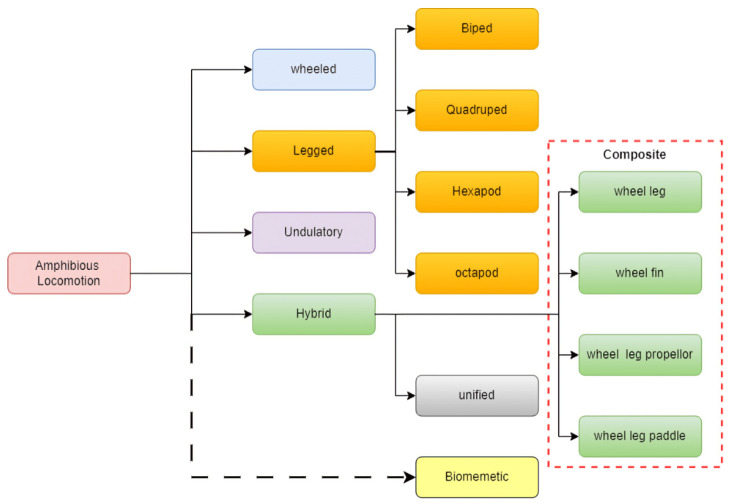
Classification of amphibious robots [3].

As shown in Figure 1, current amphibious robots typically utilize one of four locomotion methods: (1) undulatory motion, (2) legged locomotion, (3) fin-based propulsion, and (4) hybrid wheel–leg systems [1,2,4,5,6]. Representative examples include snake-inspired undulatory robots employing lateral oscillation [7], salamander-inspired legged robots capable of switching between swimming and walking gaits [2,8], fin-based robots such as the AQUA series that utilize oscillatory fins [4], and hybrid wheel–leg robots like the Whegs series designed for versatility across various terrains [9,10,11]. Recent advances in soft and biohybrid propulsion have also enabled skeletal-skin fin models for low-speed aquatic maneuvering, exemplified by biorobotic cormorant-inspired flippers with anisotropic compliant structures [12]. In parallel, emerging satellite-scale amphibious robots—such as light-driven soft microrobots that fuse inchworm-like terrestrial motion and water-strider-inspired aquatic gait—have demonstrated rapid multimodal transition and remote wireless control in milligram-scale platforms [13]. These miniaturized designs highlight promising directions in untethered amphibious actuation and terrain adaptation.

Although these designs exhibit promising multimodal capabilities, they often suffer from significant challenges. These include limited adaptability to irregular terrain, high actuator complexity, fragile mechanical designs, and poor energy efficiency during transitions between mediums [14,15]. As highlighted in recent reviews, especially in unstructured environments such as surf zones or rocky transitional areas, robots face obstacles like uneven surfaces, shoals, and slopes that demand not only robust propulsion but also mechanical compliance and real-time adaptability [16,17].

To further complicate the design, amphibious robots employing separate propulsion mechanisms for land and water modes often become bulky and complex to control [18]. Hybrid mechanisms with unified structures can simplify the system but often require intricate transformations or passive compliance to function effectively in both environments [19]. Achieving a balance between adaptability, mobility, and control simplicity remains an ongoing design challenge in the field.

**Figure 2:**
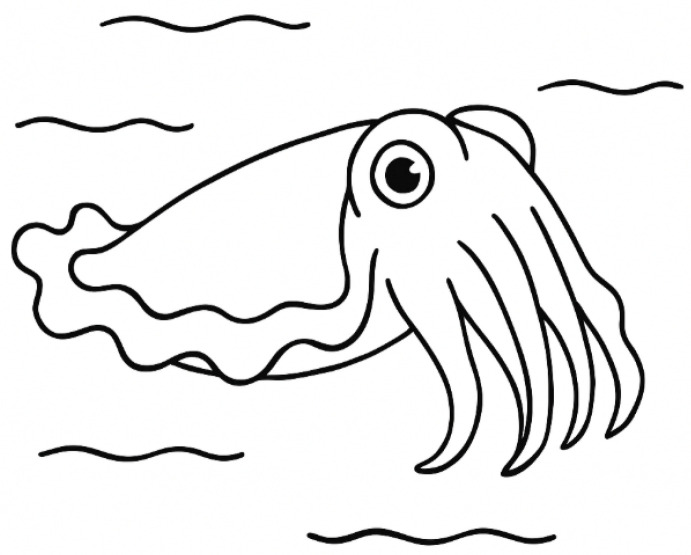
Oscillatory lateral fin movement of a cuttlefish.

**Figure 3:**
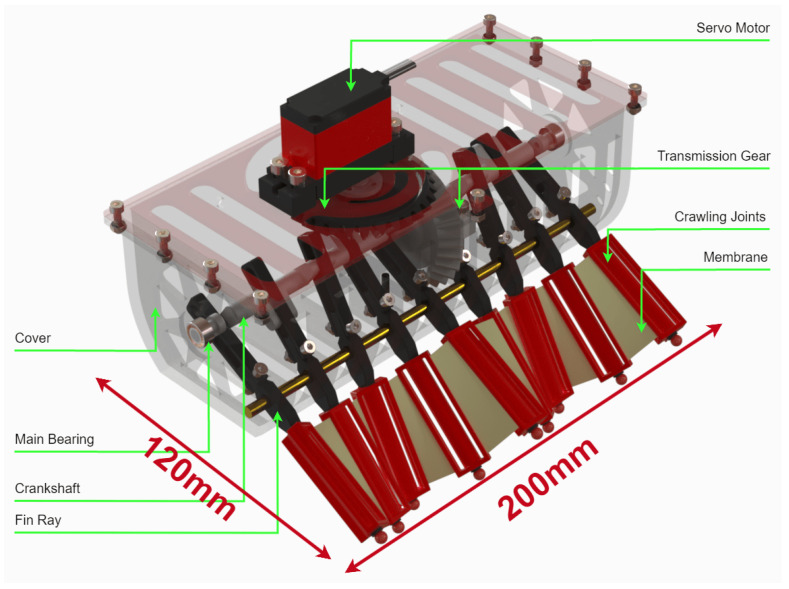
An overview of the proposed wave-like propulsion mechanism.

To address the limitations of conventional amphibious locomotion strategies, this study focuses on the principle and realization of a novel bio-inspired wave propulsion mechanism. Drawing inspiration from the undulatory fin motion of cuttlefish (Figure 2), we introduce a Crank–Slider–Rocker transmission system coupled with a rigid–flexible structural design to generate continuous traveling wave-like motion across both aquatic fins and terrestrial legs (Figure 3). While this paper presents the complete development process—including mechanical design, embedded control implementation on an ESP32 platform, and the modulation of key wave parameters such as amplitude, frequency, and direction—the central emphasis is placed on uncovering the underlying locomotion mechanism and validating its effectiveness.

Unlike previous designs such as the crank–rocker-driven robot in [20], which requires switching between separate swimming and crawling modes, our robot employs a unified Crank–Slider–Rocker mechanism and a rigid–flexible fin structure that enables continuous undulatory motion across both land and water without transformation. This simplifies the mechanical design and improves locomotion adaptability.

Extensive dynamic simulations and real-world experiments were conducted in both water and land environments to evaluate the proposed locomotion approach. The results highlight the system’s capacity for smooth and efficient movement using minimal actuation input. The rigid–flexible coupling plays a crucial role in terrain adaptability, while the unified propulsion strategy enhances control simplicity and energy performance. By systematically exploring the kinematics, implementation, and validation of this wave-based locomotion principle, this study contributes a comprehensive experimental framework that advances the development of highly adaptable amphibious robots.

By exploiting passive compliance, our design effectively mimics biomechanical principles found in nature, achieving smooth, efficient, and continuous locomotion across both aquatic and terrestrial environments.

This paper is structured as follows: Section 2 explains the locomotion principles and kinematic models for swimming and crawling. Section 3 details the materials and methods used in mechanical construction and electronic integration. Section 4 presents simulation and experimental results validating the proposed mechanism. Finally, Section 5 concludes the study and outlines directions for future work.

## 2. Locomotion Principle and Kinematic Modeling

In this section, we investigate the fundamental principles of undulatory crawling and swimming locomotion, followed by a kinematic and dynamic modeling approach to characterize the proposed system. Inspired by biological wave-like motion, we develop a Crank–Slider–Rocker mechanism that generates the required mechanical oscillations to achieve both terrestrial crawling and aquatic swimming. This mechanism leverages a rigid–flexible coupling structure, allowing the smooth propagation of wave motion while maintaining structural stability.

### 2.1. Swimming Locomotion

Undulatory swimming, characterized by traveling wave propagation along fins, is a fundamental aquatic locomotion strategy extensively observed in gymnotiform, rajiform, and amiiform swimmers [21,22]. Aquatic species adopting such Median or Paired Fin (MPF) propulsion modes typically exhibit superior maneuverability, hovering stability, and energy efficiency, particularly in low-speed and precision maneuvering scenarios [3,23,24,25].

Fish propulsion mechanisms are generally classified into two distinct modes:

- Body and Caudal Fin (BCF) Propulsion: Utilized by fast swimmers such as tunas and sharks, this mode involves lateral undulation of the body combined with rapid oscillation of the caudal fin, optimized for high-speed swimming but generally sacrificing maneuverability [23,24].
- Median and Paired Fin (MPF) Propulsion: Observed in species like the knifefish, stingrays, and cuttlefish, MPF propulsion involves wave propagation along fins positioned dorsally, ventrally, or laterally, enabling precise control, minimal hydrodynamic drag, and high efficiency at lower speeds [22,25,26,27].

The MPF propulsion mode is particularly advantageous for biomimetic robotics due to its inherent capabilities for multi-directional maneuverability and efficient thrust generation. Species such as the knifefish generate thrust by propagating undulations along their elongated anal fins, maintaining stationary body positions and reducing hydrodynamic drag [22,26]. Similarly, stingrays utilize broad pectoral fins to produce controlled undulatory waves, providing remarkable agility and efficiency [25,27].

**Figure 4:**
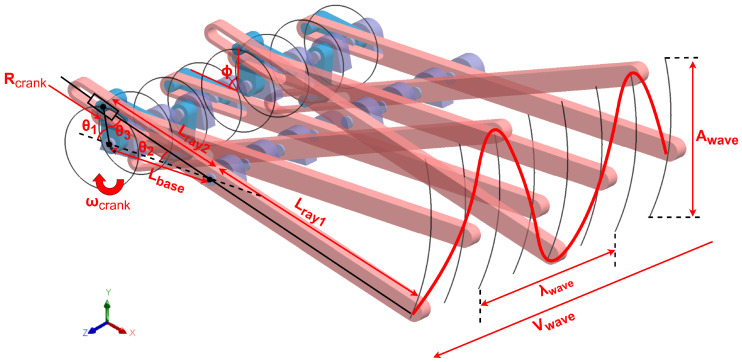
A kinematic model of the Crank–Slider–Rocker mechanism. Key parameters: $R_{\mathrm{crank}}$: crank radius; $L_{\mathrm{base}}$: fixed linkage; ${\left\{L_{\mathrm{rayi}}\right\}}_{i=1,2}$: fin ray segments; ${\left\{\theta_{i}\right\}}_{i=1,2,3}$: joint angles.

Based on the previously discussed principles of undulatory propulsion, we designed a novel Crank–Slider–Rocker mechanism to transform circular motion into periodic oscillations of the fin rays, as illustrated in Figure 4. By introducing a phase shift between multiple fin rays, this mechanism successfully generates harmonic wave-like motion along the fin array. The angular motion of each fin ray can be described by the following mathematical model:

In our proposed Crank–Slider–Rocker mechanism, the circular motion of the crank is converted into oscillatory motion of the fin rays. The key geometric transformation occurs due to the linkages, which amplify the crank displacement into larger oscillations of the fin ray tip.

For a crank rotating at angular velocity $\omega_{\mathrm{crank}}$, the instantaneous length $L_{\mathrm{ray}2}$ follows

(1) $$L_{\mathrm{ray}2}\left(\theta_{1}\right)=\sqrt{R_{\mathrm{crank}}^{2}+L_{\mathrm{base}}^{2}-2R_{\mathrm{crank}}L_{\mathrm{base}}\cos\theta_{1}},\;\theta_{1}\left(t\right)=\omega_{\mathrm{crank}}t$$

The oscillation angles $\theta_{2}$ (fin ray) and $\theta_{3}$ (closure angle) satisfy

(2) $$\begin{array}{cc} \theta_{2} & =\arcsin\left(\frac{R_{\mathrm{crank}}\sin\theta_{1}}{L_{\mathrm{ray}2}}\right)\\ \theta_{3} & =\pi -(\theta_{1}+\theta_{2}) \end{array}$$

The time-dependent ratio between fin ray segments is defined as

(3) $$K\left(t\right)≜\frac{L_{\mathrm{ray}1}}{L_{\mathrm{ray}2}\left(t\right)}=\frac{L_{\mathrm{ray}1}}{\sqrt{R_{\mathrm{crank}}^{2}+L_{\mathrm{base}}^{2}-2R_{\mathrm{crank}}L_{\mathrm{base}}\cos\theta_{1}}}$$

The maximum oscillation amplitude occurs when the closure angle satisfies $\theta_{3}=\frac{\pi }{2}+n\pi$ (n∈Z), leading to

(4) $$A_{\mathrm{wave}}=2K_{\max}R_{\mathrm{crank}},\;\mathrm{where}\;K_{\max}={\left.\frac{L_{\mathrm{ray}1}}{L_{\mathrm{ray}2}}\right|}_{\theta_{3}=\pi /2}$$

This condition corresponds to the slider reaching its extreme positions, maximizing mechanical advantage.

Sequential fin ray oscillations with phase shift $\Delta \phi_{n}$ between adjacent rays generate a traveling wave:

(5) $$y\left(x,t\right)=A_{\mathrm{wave}}\sin\left(\underset{k}{\underbrace{\frac{2\pi }{\lambda_{\mathrm{wave}}}}}x-\underset{\omega_{\mathrm{wave}}}{\underbrace{2\pi f_{\mathrm{wave}}}}t+\phi_{n}\right)$$

The wave characteristics are constrained by

(6) $$\begin{array}{cc} \lambda_{\mathrm{wave}} & =\frac{2\pi d}{\Delta \phi_{n}}\;\left(\mathrm{Structural}\right)\\ f_{\mathrm{wave}} & =\frac{\omega_{\mathrm{crank}}}{2\pi }\;\left(\mathrm{Kinematic}\right)\\ V_{\mathrm{wave}} & =\lambda_{\mathrm{wave}}f_{\mathrm{wave}}=\frac{d\omega_{\mathrm{crank}}}{\Delta \phi_{n}} \end{array}$$

where *d* is the fixed spacing between fin rays.

The actual swimming velocity incorporates hydrodynamic efficiency η:

(7) $$V_{\mathrm{sw}}=\eta V_{\mathrm{wave}}=\eta \frac{d\omega_{\mathrm{crank}}}{\Delta \phi_{n}}$$

The mechanism’s performance is governed by three adjustable parameters:

- Wave Amplitude: Controlled via the crank radius $R_{crank}$ through Equation (4).
- Wave Frequency: Determined by $\omega_{crank}$ (Equation (6)).
- Wave Direction: Reversed by setting $\Delta \phi_{n}<0$.

In this study, η is treated as an empirical coefficient, obtained via the linear regression of experimental velocity measurements under varied crank speeds. It incorporates unmodeled hydrodynamic drag, fin–body interaction effects, and viscous losses. While this linear approximation is valid in the tested low-to-moderate Reynolds number regime, we acknowledge that η may become nonlinear and dependent on $\omega_{\mathrm{crank}}$ or fin geometry under higher flow speeds or altered configurations.

Based on the above analyses, a comprehensive theoretical framework is established, elucidating the inherent relationships from rotational motion to fin-ray undulations and thrust generation. Specifically, the kinematic foundations (Equations (1) and (3)) reveal how the Crank–Slider–Rocker mechanism geometrically converts rotary motion into fin-ray oscillations. The wave amplitude generation model (Equation (4)) explicitly relates the crank radius to the maximum oscillation amplitude. Wave propagation dynamics (Equations (5) and (6)) characterize the relationships among wavelength, frequency, and structural parameters. Lastly, the thrust conversion model (Equation (7)) quantitatively accounts for hydrodynamic drag and energy losses.

Overall, this theoretical framework provides a robust foundation for optimizing amphibious robot locomotion, enabling the independent tuning of wave amplitude, propagation frequency, and phase differences to effectively balance swimming velocity and maneuverability.

### 2.2. Crawling Locomotion

Undulatory crawling is a widely adopted locomotion strategy observed in nature, notably among serpentine and limbless organisms such as snakes, worms, and caterpillars, which propagate traveling waves along their bodies to achieve forward thrust [28,29,30]. Inspired by these biological organisms, robotics research has successfully adapted similar wave-based propulsion methods for terrestrial locomotion. A prominent example is the Single Actuator Wave-like (SAW) robot developed by Zarrouk et al. [28], which utilizes a single rotating actuator to create continuous wave propagation across its rigid body segments. Despite its efficiency, the SAW robot encounters mechanical constraints and reduced adaptability on uneven surfaces due to rigid linkages.

Rigidly linked wave robots often exhibit challenges such as limited adaptability, increased mechanical complexity, and structural fragility on irregular terrain conditions [29,30]. To overcome these limitations, this study introduces a novel Crank–Slider–Rocker mechanism featuring rigid–flexible coupling. Unlike traditional rigid designs, our approach employs elastic membranes to connect rigid segments, significantly enhancing the robot’s ability to conform adaptively to terrain irregularities and smoothly propagate waves throughout its structure.

**Figure 5:**
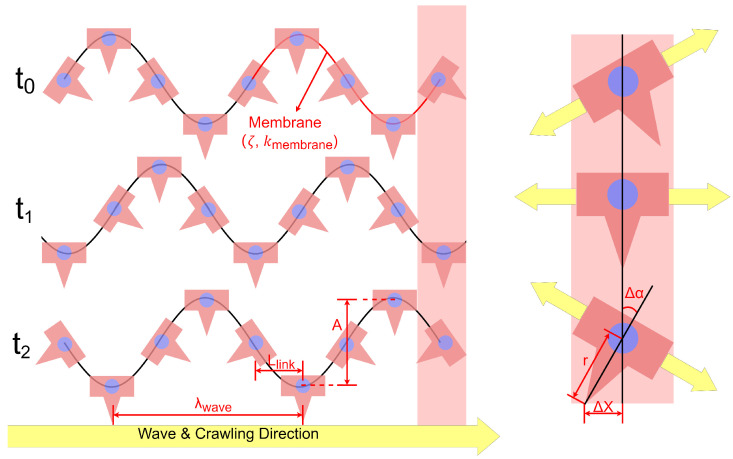
Kinematic model and wave propagation in the crawling mechanism. Key parameters: *A*: wave amplitude; $\lambda_{\mathrm{wave}}$: wavelength; $L_{\mathrm{link}}$: link length; ζ: flexibility factor; $k_{\mathrm{membrane}}$: membrane stiffness; Δα: joint rotation angle; *r*: tip radius; ΔX: horizontal displacement.

Figure 5 illustrates the Crank–Slider–Rocker mechanism and wave propagation dynamics. The flexible membranes (characterized by flexibility parameters and stiffness) enable segments to rotate passively in response to ground contact forces. At each time step, a sinusoidal wave profile characterized by amplitude, wavelength, and link spacing propagates along the robot’s body from head to tail. Adjacent segments oscillate with alternating phases, forming a coordinated traveling wave.

The traveling wave motion can be mathematically described by

(8) $$y\left(x,t\right)=A\sin\left(\frac{2\pi }{\lambda_{\mathrm{wave}}}x-2\pi ft\right)$$

where the amplitude directly corresponds to the vertical displacement magnitude, and the wavelength denotes the distance between wave peaks. The wave propagation velocity is given by the product of wavelength and frequency:

(9) $$V_{\mathrm{wave}}=\lambda_{\mathrm{wave}}\cdot f$$

The actual forward crawling speed depends on how efficiently this wave motion translates into linear displacement. Following the advance ratio (AR) model in [28], we have:

(10) $$V_{\mathrm{robot}}=\mathrm{AR}\cdot V_{\mathrm{wave}}=\mathrm{AR}\cdot \lambda_{\mathrm{wave}}f$$

The AR is influenced by the curvature radius at the segment tip and wave amplitude, expressed as

(11) $$\mathrm{AR}=\frac{2\pi rA}{\lambda_{\mathrm{wave}}^{2}}$$

As further detailed in Figure 5, each segment’s rotational displacement is passively driven by the elastic deformation of the membrane, characterized by membrane stiffness:

(12) $$\Delta \alpha \approx \frac{k_{\mathrm{membrane}}A}{\lambda_{\mathrm{wave}}}$$

This rotational displacement, combined with the tip curvature radius, translates into horizontal displacement:

(13) ΔX≈rΔα

In summary, the proposed crawling mechanism integrates mechanical wave generation, flexible joint deformation, and thrust conversion into a unified theoretical framework. The wave dynamics (Equations (8) and (9)) govern the propagation of traveling waves along the robot body, defining the amplitude, frequency, and wavelength parameters critical for effective locomotion. Meanwhile, the efficiency of converting these waves into forward displacement is quantified by the advance ratio (AR) model (Equations (10) and (11)), which incorporates the compliance characteristics of the elastic membranes. Additionally, the kinematic relationships (Equations (12) and (13)) highlight how passive elastic deformation directly contributes to rotational and translational motion.

## 3. Materials and Methods

To comprehensively evaluate the effectiveness of the proposed wave-motion propulsion mechanism, we conducted both dynamic simulations and physical experiments under swimming and crawling conditions. The validation process was divided into two stages: first, a simulation was performed based on the developed kinematic model to verify the feasibility and coherence of wave propagation across the robot’s limbs; second, a series of controlled experiments were carried out using a physical prototype to assess real-world locomotion performance.

In the simulation phase, time-resolved wave propagation and leg displacement trajectories were analyzed to ensure that the Crank–Slider–Rocker mechanism with rigid–flexible coupling could generate stable, continuous traveling waves. Subsequently, physical experiments were conducted to quantify the robot’s locomotion performance, including its straight-line displacement speed and in-place rotational capability. These experiments relied on a monocular camera system for visual tracking, an onboard IMU for angular motion measurement, and photointerrupters for event-based timing.

The combined simulation and experimental results confirm the feasibility, controllability, and versatility of the proposed propulsion mechanism in both aquatic and terrestrial environments.

### 3.1. Simulation of Wave-like Propulsion

To verify the effectiveness of the kinematic model and assess the feasibility of the proposed Crank–Slider–Rocker mechanism, a dynamic simulation was carried out to visualize the time-dependent wave propagation along the robot’s limbs. The model parameters were based on the mechanical design and equations detailed in Section 2, incorporating flexible link deformation, phase shift, and crank-driven oscillations.

**Figure 6:**
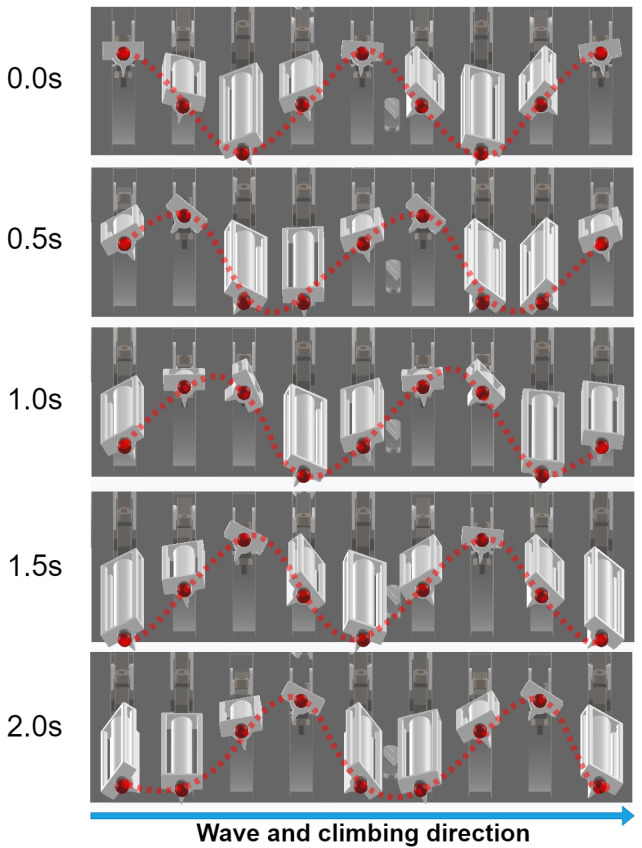
A timeline of the motion of the wave-like propulsion.

The simulation focused on both qualitative wave propagation and quantitative leg displacement analysis over a complete motion cycle. Figure 6 illustrates the spatiotemporal evolution of the leg trajectories from t=0s to t=2.0s, showing a continuous traveling wave propagating from left to right. The red dotted line indicates the vertical position of each limb tip at successive time intervals, and clearly demonstrates the desired phase-shifted oscillatory pattern.

**Figure 7:**
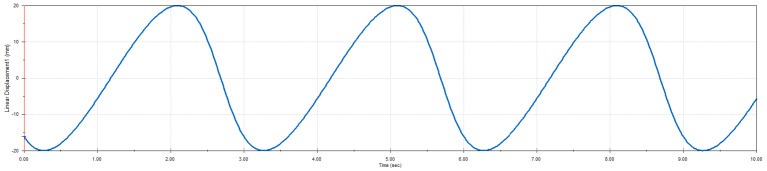
Displacement of the end of legs.

Figure 7 presents the vertical displacement of a representative leg tip over time, extracted from the simulation output. The waveform confirms harmonic motion consistent with the sinusoidal input defined in the model:

$$y\left(t\right)=A\sin\left(2\pi ft+\phi_{n}\right)$$

where *A* is the amplitude, *f* is the frequency, and $\phi_{n}$ is the phase shift. The periodicity and smoothness of the trajectory validate the correctness of the mechanical transmission and phase control strategy.

These simulation results confirm that the proposed rigid–flexible coupling design successfully generates continuous traveling waves with tunable parameters. The consistency between model predictions and simulated leg trajectories provides strong evidence for the mechanical plausibility of the wave-like propulsion mechanism prior to physical prototyping.

### 3.2. Experimental Setup

**Figure 8:**
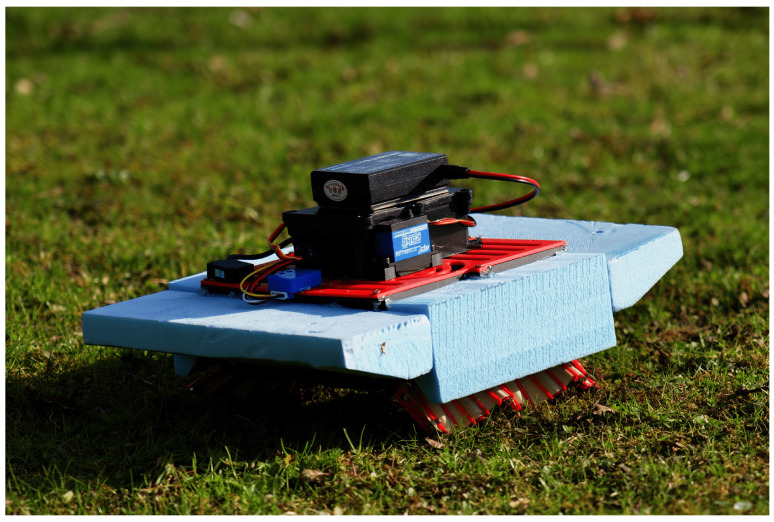
The crawling prototype on grass.

**Figure 9:**
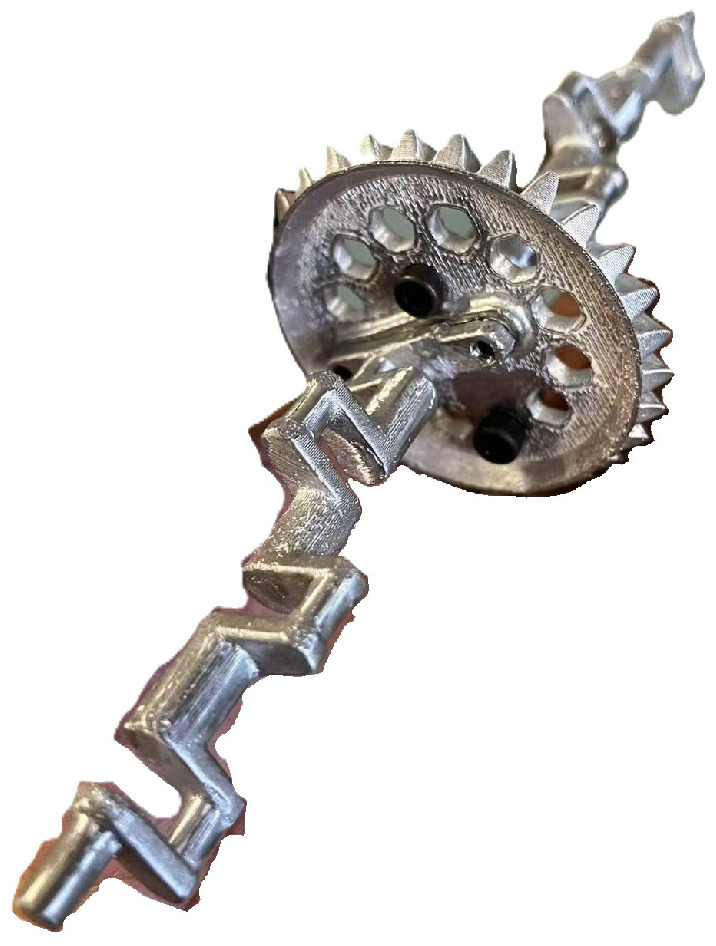
Zinc-casted crankshaft.

The prototype was primarily constructed using polylactic acid (PLA) material through fused deposition modeling (FDM) 3D printing, chosen for its suitability in rapid prototyping and adequate strength-to-weight ratio (Figure 8). PLA’s ease of processing and biodegradability also align with sustainable development goals. To reduce friction and improve wear resistance in the high-cycle transmission system, the crank components were manufactured via zinc alloy die casting, leveraging the material’s low surface friction coefficient and durability in repetitive mechanical contact, as shown in Figure 9.

**Figure 10:**
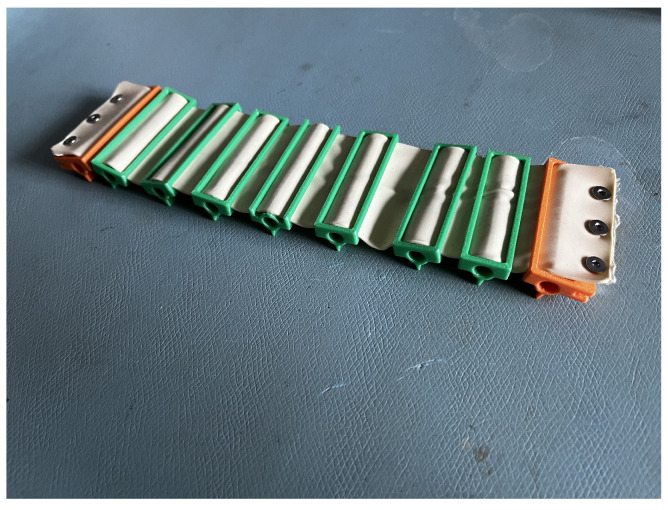
Latex-casted membrane.

The flexible membrane joints between rigid link segments were fabricated using latex rubber, selected for its high elasticity and tear resistance. Custom 3D-printed molds were designed and printed to form the membranes, with a uniform thickness of 1 mm, ensuring repeatability and mechanical consistency. This molding process allowed tight control over geometry while preserving compliance essential for wave propagation. The membranes were bonded to the rigid body using flexible adhesive to allow passive joint deformation under dynamic loading, as illustrated in Figure 10.

**Figure 11:**
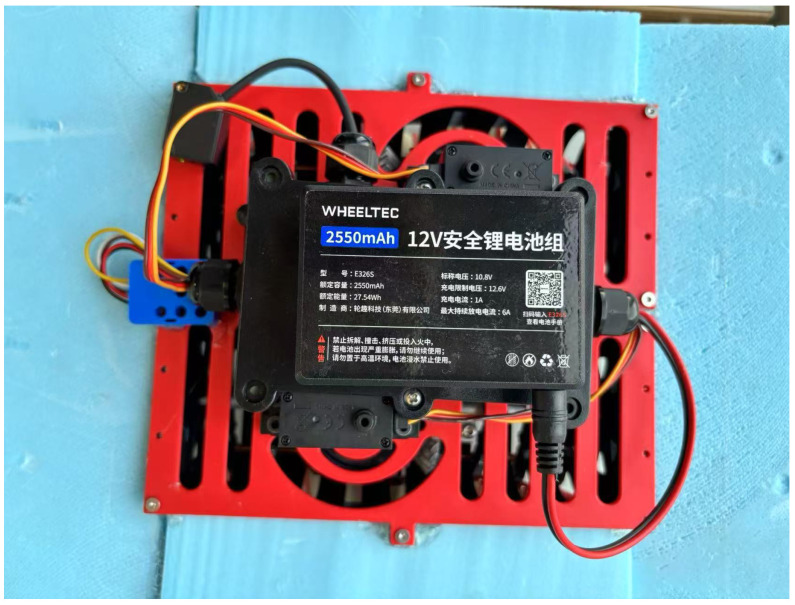
Prototype electronics system with waterproof housing.

For the electronic system, all sensors and actuators were connected via conventional jumper wires to a general-purpose ESP32 expansion board, eliminating the need for custom PCB fabrication. The board integrated power distribution and GPIO breakout functionalities, facilitating straightforward development and debugging. To ensure safe operation in aquatic environments, the entire electronic assembly was enclosed within a waterproof sealed box. This protective housing enabled stable underwater performance while maintaining accessibility for maintenance and updates, as shown in Figure 11.

To quantitatively assess steady-state straight-line locomotion, controlled experiments were conducted in both terrestrial and aquatic environments using calibrated measurement tracks. For crawling trials, the robot traversed a flat surface bounded by two parallel guide rails. The test path was marked at 500 mm intervals over a total length of 1000mm. A top-mounted monocular camera operating at 30fps continuously recorded the motion. To eliminate transient effects, the robot was allowed to reach a constant speed before entering the measurement region.

For aquatic trials, the robot was deployed in a narrow test channel measuring 250mm in length. The same camera system was positioned laterally to capture the robot’s progression across the waterway. Frame-by-frame position tracking was performed to determine the effective swimming velocity once the robot achieved consistent forward movement.

For in-place rotation, the angular velocity was recorded using the onboard IMU (MPU6050, TDK InvenSense, Boston, MA, USA). The yaw angle over time was used to compute the average rotation speed in degrees per second.

## 4. Results and Discussion

### 4.1. Swimming Performance

To evaluate the real-world aquatic locomotion capability of the prototype, the fin modules were actuated at a wave frequency of 1.25Hz (i.e., period of 0.8s) with a phase shift of $\Delta \phi =90^{\circ }$ between adjacent rays. This configuration corresponds to a wave propagation speed of

$$V_{\mathrm{wave}}=\frac{d\cdot \omega_{\mathrm{crank}}}{\Delta \phi_{n}}=\frac{50\cdot 2\pi \cdot 1.25}{\pi /2}=160\;\mathrm{mm}/s$$

Experimental evaluation was performed in a test tank with transparent sides for video analysis. Using calibrated ground markers, the swimming velocity was measured to be approximately 12.5mm/s. This result agrees with the theoretical estimation using the hydrodynamic efficiency factor:

$$V_{\mathrm{sw}}=\eta V_{\mathrm{wave}}=0.078125\times 160=12.5\;\mathrm{mm}/s$$

**Figure 12:**
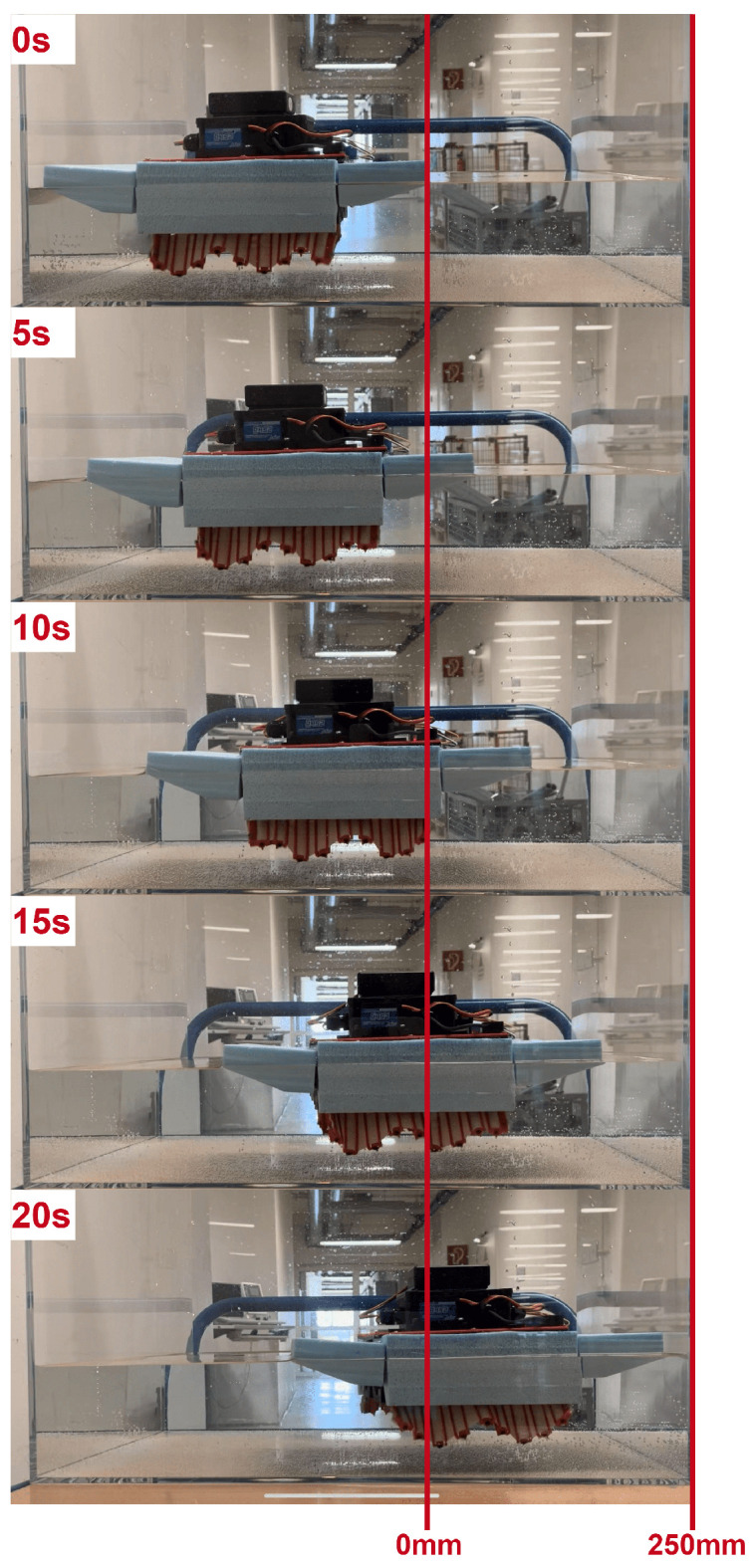
Snapshot sequence of swimming test at 20s intervals under water.

**Figure 13:**
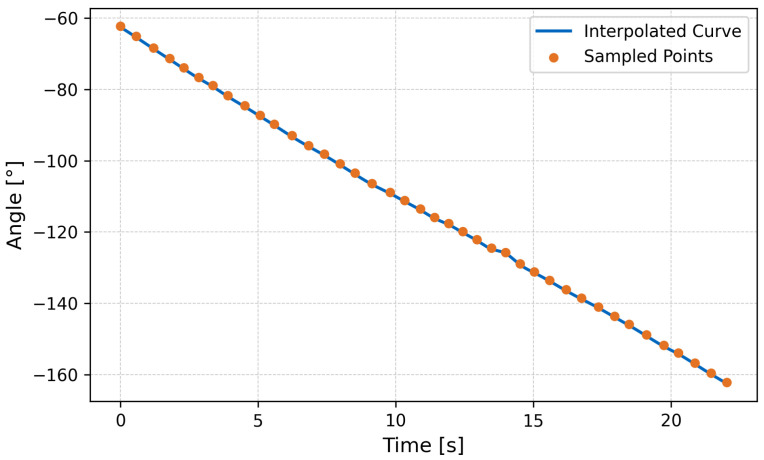
Angular position on water over time.

**Figure 14:**
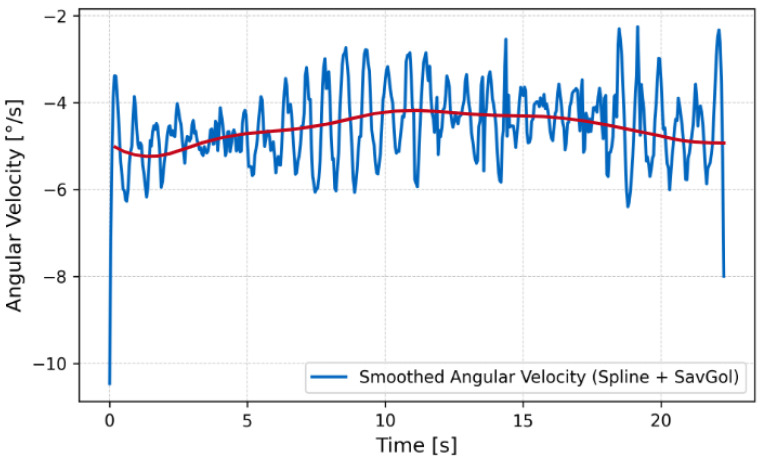
Angular velocity on water over time. Here, the red line indicates the smoothed angular velocity.

The close match between the model and experimental measurements supports the validity of the wave propulsion framework derived in Equation (7). The robot maintained neutral buoyancy and demonstrated stable forward propulsion with low pitch oscillations (Figure 12, Figure 13 and Figure 14).

The coefficient η was obtained through linear regression of experimental data collected at various crank speeds. No computational hydrodynamic model was employed. The resulting η represents an averaged empirical fit under the tested conditions.

In-place turning was achieved by reversing the wave propagation direction on the left fin while maintaining the forward wave on the right fin. The IMU recorded an average yaw rate of $5^{\circ }/s$ during this maneuver, confirming effective directional control.

### 4.2. Crawling Performance

For terrestrial locomotion, identical wave parameters were applied to the leg segments to achieve crawling motion. Based on the derived kinematic model using a wave amplitude of A=40mm, radius r=5mm, and wavelength λ=100mm, the theoretical advance ratio was calculated to be AR≈0.126. Given a wave propagation speed of $V_{\mathrm{wave}}=160\;\mathrm{mm}/s$ (based on actuation frequency), the estimated forward speed becomes

$$\begin{array}{cc} AR & =\frac{2\pi rA}{\lambda^{2}}=\frac{2\pi \cdot 5\;\mathrm{mm}\cdot 40\;\mathrm{mm}}{{\left(100\;\mathrm{mm}\right)}^{2}}\approx \;0.126\\ V_{\mathrm{robot}} & =AR\cdot V_{\mathrm{wave}}=0.126\times 160=20.2\;\mathrm{mm}/s \end{array}$$

**Figure 15:**
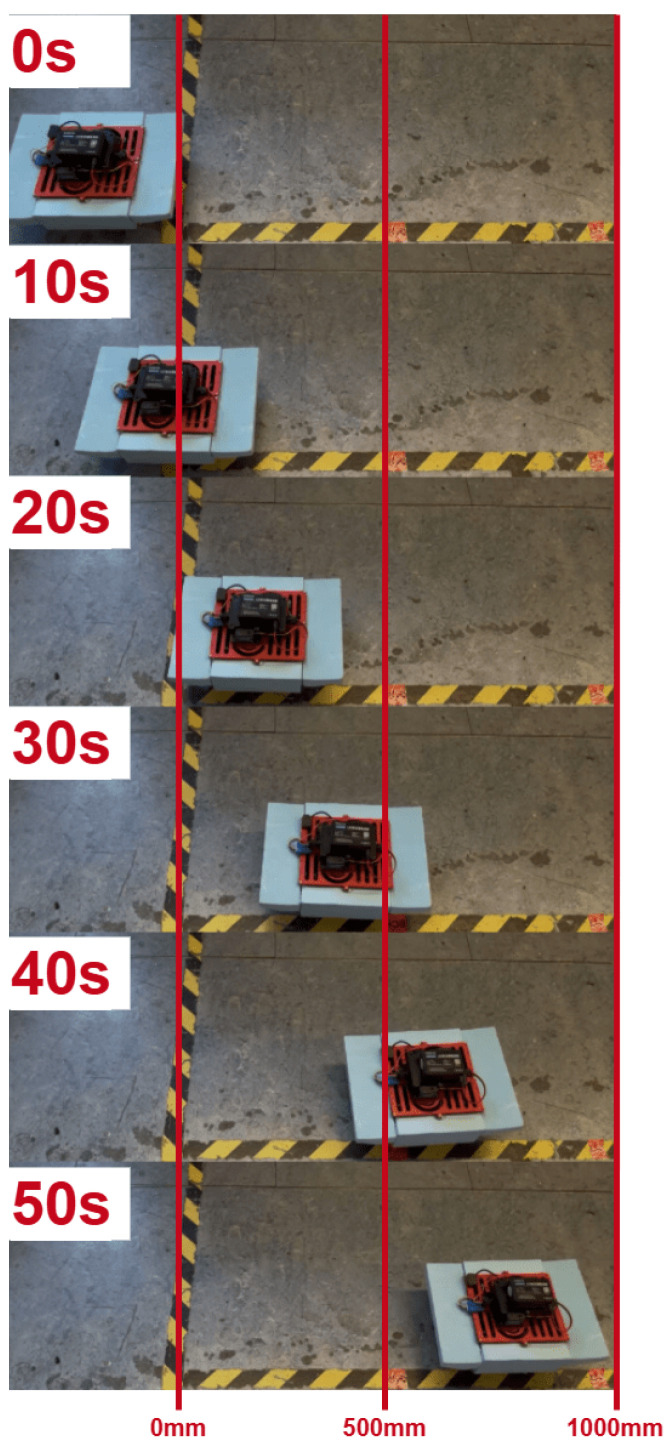
Snapshot sequence of crawling test over surface at 50s intervals.

**Figure 16:**
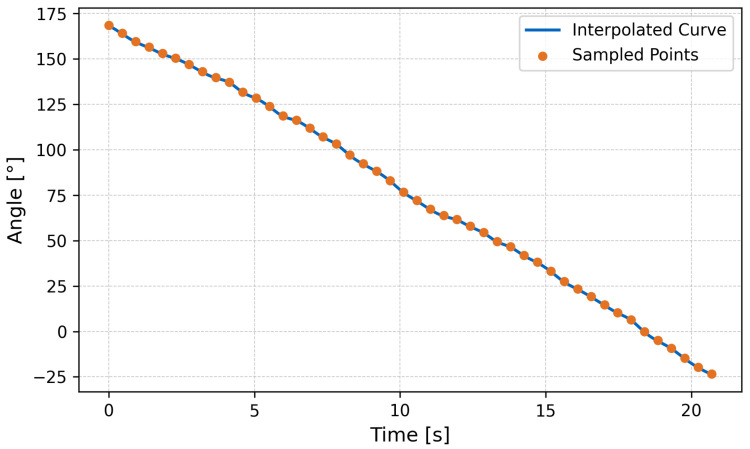
Angular position over time.

**Figure 17:**
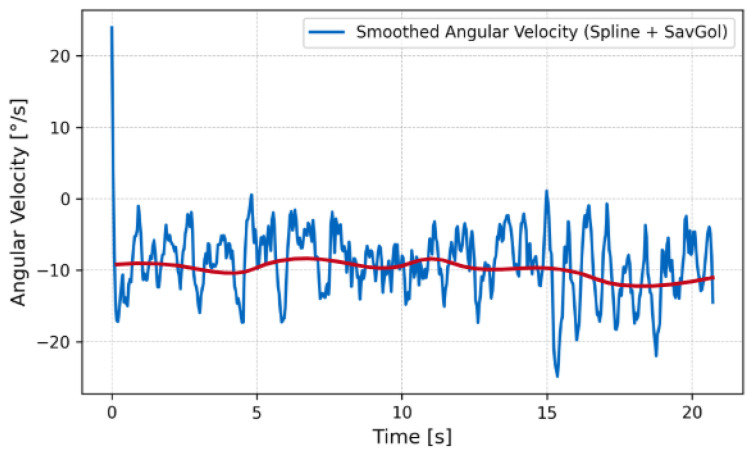
Angular velocity over time. Here, the red line indicates the smoothed angular velocity.

In practical crawling experiments (shown in Figure 15), the robot’s forward velocity was measured using side-mounted camera tracking and calibrated ground markers. The observed straight-line speed was approximately 20mm/s (0.1BL/s), which is in strong agreement with the predicted value, thereby validating the accuracy of the kinematic model for land-based crawling. This result demonstrates that the proposed wave-based leg actuation mechanism can produce consistent and predictable locomotion performance on solid ground (Figure 16 and Figure 17). The fin–ground interaction exhibits anisotropic friction behavior, where forward slippage is minimized during the power stroke. Sliding is allowed to a limited extent but does not dominate the propulsion mechanism.

Additionally, in-place rotation was achieved by introducing phase asymmetry between the left and right leg arrays. The onboard IMU recorded an average rotational velocity of $10^{\circ }/s$, confirming the robot’s ability to maneuver directionally in place.

### 4.3. Discussion

The experimental results affirm the feasibility and functional robustness of the proposed wave-based propulsion system across both water and land environments. The observed locomotion behavior is in close agreement with the theoretical model derived from the Crank–Slider–Rocker mechanism and the corresponding kinematic equations.

In aquatic settings, the measured swimming velocity of 12.5mm/s corresponds closely to the predicted value based on the applied wave frequency and calculated hydrodynamic efficiency. The robot exhibited consistent directional motion with minimal pitch oscillation, highlighting the advantages of using biologically inspired undulatory waveforms for stable aquatic locomotion. The in-place turning capability, validated by an average yaw rate of $5^{\circ }/s$, further demonstrated precise maneuverability using phase-modulated wave control.

However, the relatively low swimming speed suggests opportunities for further optimization. One strategy is to increase the actuation frequency of the crank input to raise the overall wave propagation speed. Additionally, extending the length of the fin rays could amplify the effective amplitude of oscillation, resulting in greater thrust generation. Increasing the surface area of the fin membrane may also improve hydrodynamic interaction and propulsion efficiency. Alternative material selections for the fin structure could reduce drag or enhance stiffness in key directions, allowing for more effective energy transfer from mechanical input to fluid displacement. These improvements could collectively contribute to a substantial boost in underwater performance.

On land, the crawling performance also aligned with theoretical predictions. The measured forward velocity of 20mm/s closely matched the calculated value using the advance ratio and wave propagation speed. This agreement validates the effectiveness of the kinematic model even under friction-dominant conditions. The robot successfully demonstrated in-place turning on flat terrain with an angular velocity of $10^{\circ }/s$, confirming the applicability of wave-phase control in terrestrial locomotion as well.

Overall, these results support the conclusion that the rigid–flexible coupled transmission system enables predictable and controllable locomotion through tunable waveform parameters. The successful integration of simulation and experimental outcomes reinforces the value of the proposed design framework for future amphibious robotic applications. Further improvements could focus on optimizing hydrodynamic efficiency, improving surface interaction during crawling, extending sensor feedback integration, and exploring multi-degree-of-freedom actuation schemes for enhanced agility and adaptability.

## 5. Conclusions

This study presents a novel mechanical architecture for amphibious robotic propulsion, featuring a Crank–Slider–Rocker mechanism integrated with a rigid–flexible coupling configuration. The proposed system enables biomimetic undulatory locomotion through a single-degree-of-freedom rotary input, achieving continuous wave propagation along articulated limbs without requiring complex multi-actuator coordination.

A comprehensive kinematic model was derived to capture the geometric and temporal transformation from crank motion to fin-ray oscillation, enabling the analytical tuning of critical wave parameters such as amplitude, wavelength, frequency, and propagation direction. This framework not only guided the physical design but also allowed predictive analysis of propulsion performance across aquatic and terrestrial environments.

Time-resolved dynamic simulations verified that the mechanical structure supports coherent phase propagation and stable traveling wave generation. The simulated trajectories confirmed the harmonic nature of oscillation and validated the phase modulation strategy embedded in the Crank–Slider–Rocker mechanism.

Physical experiments were conducted in both water and land testbeds under controlled conditions. The measured swimming speed of 12.5mm/s and crawling speed of 20mm/s closely matched theoretical predictions derived from the model, confirming its accuracy and robustness. Angular velocity tracking during turning maneuvers further demonstrated the feasibility of phase-based direction control. The close agreement across modeling, simulation, and empirical validation reinforces the mechanical and kinematic viability of the proposed approach.

Compared to conventional robotic propulsion systems, this architecture achieves functionally rich wave-based motion with significantly reduced actuator complexity. The system offers promising scalability and modularity, laying the groundwork for high-efficiency, low-cost amphibious locomotion platforms. Future work will focus on enhancing control autonomy, integrating soft sensor feedback for adaptive gait modulation, and extending the approach to three-dimensional fin actuation for improved hydrodynamic maneuverability.

## Data Availability

The original contributions presented in this study are included in the article/Supplementary Materials. Further inquiries can be directed to the corresponding author.

## Supplementary Materials

The following supporting information can be downloaded at: https://www.mdpi.com/article/10.3390/biomimetics10060396/s1, Video S1: Crawling and swimming motions of the cuttlefish-inspired robot.
